# Spontaneous symmetry breaking in persistent currents of spinor polaritons

**DOI:** 10.1038/s41598-021-01812-3

**Published:** 2021-11-17

**Authors:** Evgeny Sedov, Sergey Arakelian, Alexey Kavokin

**Affiliations:** 1 Key Laboratory for Quantum Materials of Zhejiang Province, School of Science, Westlake University, 18 Shilongshan Road, Hangzhou, 310024 Zhejiang Province China; 2 Institute of Natural Sciences, Westlake Institute for Advanced Study, 18 Shilongshan Road, Hangzhou, 310024 Zhejiang Province China; 3 Department of Physics and Applied Mathematics, Vladimir State University Named After A. G. and N. G. Stoletovs, Gorky str. 87, Vladimir, Russia 600000; 4 Spin Optics Laboratory, St. Petersburg State University, Ul’anovskaya 1, Peterhof, St. Petersburg, Russia 198504; 5 Russian Quantum Center, Skolkovo, Moscow, Russia 143025

**Keywords:** Polaritons, Polaritons

## Abstract

We predict the spontaneous symmetry breaking in a spinor Bose–Einstein condensate of exciton-polaritons (polaritons) caused by the coupling of its spin and orbital degrees of freedom. We study a polariton condensate trapped in a ring-shaped effective potential with a broken rotational symmetry. We propose a realistic scheme of generating controllable spinor azimuthal persistent currents of polaritons in the trap under the continuous wave optical pump. We propose a new type of half-quantum circulating states in a spinor system characterized by azimuthal currents in both circular polarizations and a vortex in only one of the polarizations. The spontaneous symmetry breaking in the spinor polariton condensate that consists in the switching from co-winding to opposite-winding currents in opposite spin states is revealed. It is characterized by the change of the average orbital angular momentum of the condensate from zero to non-zero values. The radial displacement of the pump spot and the polarization of the pump act as the control parameters. The considered system exhibits a fundamental similarity to a superconducting flux qubit, which makes it highly promising for applications in quantum computing.

## Introduction

Due to their wide application prospects^1–3^, the effects caused by the spin-orbit interaction (SOI) are among the most popular topics of study in various branches of contemporary physics including solid state physics^4^, optics^5,6^, ultracold atoms^7–9^, two-dimensional materials^10,11^, etc. A convenient platform for studying SOI is polaritonic systems, semiconductor heterostructures which provide conditions for hybridization of light and elementary excitations of medium^12^. The structures represent planar optical microcavities which confine light in one spatial dimension. Light strongly couples to excitons in quantum wells (QWs) embedded in the microcavities forming new hybrid eigenmodes exciton-polaritons (polaritons for brevity). The spin (pseudospin) degree of freedom is inherited by polaritons both from photon and exciton constituents. Heavy-hole excitons characterized by a projection of the angular momentum on the QW growth axis $\pm 1$ couple with photons of right- and left-circular polarizations, respectively^13,14^, forming bipartite polariton states obeying physics of spin-$\frac{1}{2}$ particles.

In atomic condensates, inducing SOI of particles is a non-trivial research problem which was solved recently by dressing atomic spin states with a pair of Raman lasers^7–9^. In contrast to atomic condensates, SOI is an inherent property of polariton condensates originated from various effects including the TE-TM splitting of photonic modes^15^, long-range exchange interaction of charge carriers^16^, controllable magneto-induced splitting in exciton pseudospin states^17–19^, etc.

According to the makeshift classification given in^9,20^, one can distinguish two types of SOI. The first type is the spin-linear-momentum interaction (SLMI) which is responsible for coupling the spin of polaritons to their momentum (or quasimomentum ${\mathbf {k}}$) that characterizes spreading of quasiparticles in the cavity plane. Among the effects caused by SLMI are the optical spin Hall effect^21–23^, the *zitterbewegung* (trembling motion) of polaritons^24,25^, polarization multistability^26^, parametric spin effects^27^ and many others^28–31^. The second type of SOI is the spin-orbital-angular-momentum interaction (SOAMI) which links the spin with the intrinsic (independent of the coordinate origin) orbital angular momentum (OAM) of particles. The most remarkable manifestations of this are spin-to-orbital angular momentum conversion^32^ and generation of half-quantum circulation states^33^ including half-vortices^34,35^.

Reduction of the problem from two-dimensional to one-dimensional^36–39^ enhances the role of nonlinearity induced by interactions and gives rise to various topological effects. The trapping of polaritons in quasi-one-dimensional ring-shaped potentials is of particular interest here^33,40,41^. In the case of a narrow ring confining potential where the radial degree of freedom is suppressed, SLMI and SOAMI problems merge.

In this manuscript, we consider evolution of a spinor exciton-polariton condensate emerging under the spatially-localized non-resonant optical pump in a cylindrical micropillar. Schematic of the possible experiment is shown in Fig. 1. The pump excites the reservoir of incoherent excitons near the center of the pillar. The reservoir feeds the polariton state via stimulated scattering processes and acts as a repulsive potential for the polariton condensate forming the ring-shaped trap together with the stationary potential created by the edges of the pillar. In detail, this excitation scheme is described in^42–44^.

The displacement of the pump spot from the center of the pillar breaks the rotational symmetry of the system. Behavior of a scalar polariton field in such potential has been investigated both experimentally and theoretically in^42–44^. It has been shown that breaking the symmetry itself does not lead to formation of azimuthal polariton currents. For the emergence of the currents, an additional factor is required which endows the system with chirality. The spin-orbit interaction of polaritons can successfully act as such factor. Here we consider SOI originated from the TE-TM splitting of polariton modes^15^ which is the most prominent effect in polariton microcavities.

The exciton-polariton condensate possesses a driven-dissipative nature. It exists under the optical pump which compensates dissipation due to a finite polariton lifetime. Thermalization time for polaritons is estimated as 1–10 ps. When the polariton lifetime is less than these estimations, polaritons never thermalize, and the condensate forms under the pump spot. We consider the polariton lifetime of about 45 ps which allows polaritons to occupy the minimum of the effective potential. As demonstrated in the experimental works^45–47^, the further increase in the polariton lifetime leads to the thermalization of polaritons.

## Results

**Figure 1:**
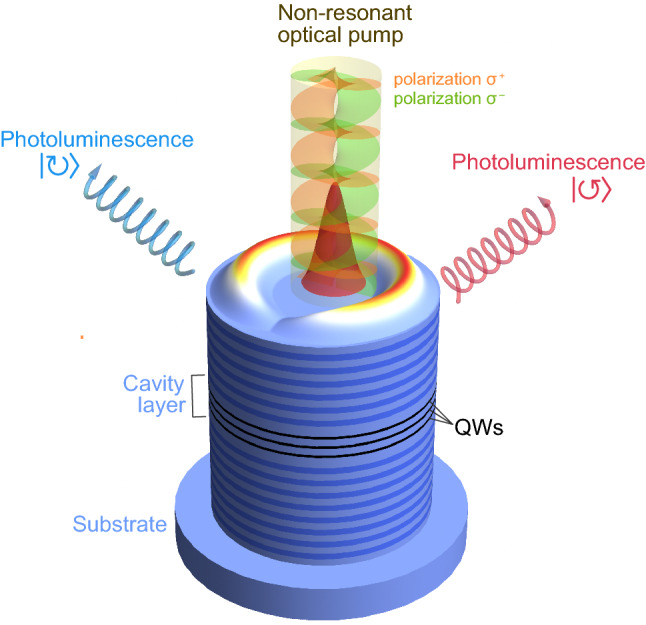
Schematic of the excitation of a spinor polariton current state in a cylindrical micropillar. Polaritons are excited by the polarized non-resonant cw optical pump slightly shifted from the center of the micropillar. Clockwise ($| \circlearrowright \rangle$) and anti-clockwise ($| \circlearrowleft \rangle$) polariton currents characterized by nonzero OAM can emerge in different polarizations. The orbital angular momentum is transferred to the photoluminescence of the condensate.

### The polariton condensate in a ring trap

The 1D single-particle Hamiltonian for the spinor exciton-polariton condensate in the ring-shaped trap can be written as follows:

1 $$\begin{aligned} {\hat{H}} _0 = \frac{\hbar ^2 {\hat{L}}^2 }{2M \rho ^2} {\check{\sigma }}_0 - \frac{\hbar \Delta }{2} \left( \frac{1}{\rho _1 ^2} + \frac{{\hat{L}}^2}{\rho ^2} \right) \left[ \cos (2 \theta ) {\check{\sigma }}_x + \sin (2 \theta ) {\check{\sigma }}_y \right] - \text {i}\frac{\hbar \Delta {\hat{L}}}{\rho _2^2} \left[ \sin (2 \theta ) {\check{\sigma }}_x - \cos (2 \theta ) {\check{\sigma }}_y \right] . \end{aligned}$$

The Hamiltonian (1) is written in the basis $\left[ \Psi _+(\theta ), \Psi _- (\theta ) \right] ^{\text {T}}$, where $\Psi _{\pm } (\theta )$ are the azimuthal wave functions of the right- (“$+$”) and left-circularly (“−”) polarized polaritons. The first term in Eq. (1) is responsible for the azimuthal kinetic energy. ${\hat{L}} = -\text {i} \partial _{\theta }$ is the OAM operator, *M* is the effective mass of polaritons in the pillar. $\rho$ is the weighted radius of the ring trap defined as $\rho ^{-2} = \langle r^{-2} \rangle _r$, where $\langle ... \rangle _r$ is averaging over the radial coordinate. $(r,\theta )$ are the polar coordinates. The second and the third terms describe the SOI effect due to the TE-TM splitting. $\Delta$ is the splitting constant. The first term in the parentheses appears as a result of spatial quantization of the radial condensate mode and it does not affect OAM directly. The dimensional constants $\rho _{1,2}$ appear as a result of averaging the directionally-anisotropic distribution of the TE-TM splitting over the radial coordinate: $\rho _1^{-2} = \langle \partial _{rr}^2 - r^{-1} \partial _r \rangle _r$, $\rho _2^{-2} = \rho ^{-2} - \langle r^{-1} \partial _r \rangle _r$. The Pauli matrices ${\check{\sigma }}_{x,y,z}$ are responsible for the spin (polarization) degree of freedom of polaritons. ${\check{\sigma }}_0$ is the $2 \times 2$ identity matrix

**Figure 2:**
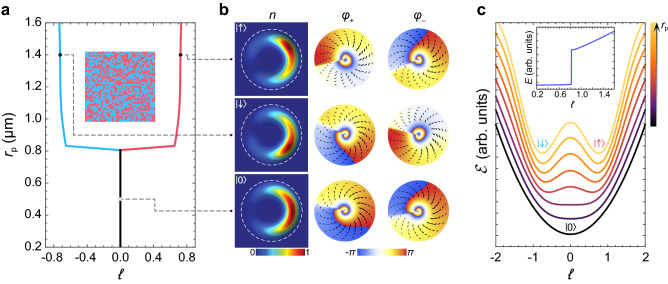
Spontaneous symmetry breaking of the polariton current state under SOI. (**a**) Variation of the average OAM per particle, $\ell$, of the exciton-polariton condensate with the displacement of the pump spot from the center of the pillar, $r_{\text {p}}$, under the linearly-polarized non-resonant optical pump ($p=0$). The insert shows results of a series of numerical experiments at $r_{\text {p}} = 1.4 \, \upmu \text {m}$. Each pixel corresponds to a separate numerical experiment. Pink and blue colors correspond to the states $| \uparrow \rangle$ and $| \downarrow \rangle$, respectively. (**b**) Spatial distribution of the density of the polariton condensate, *n* (left), as well as of the phase of the left-circularly, $\varphi _{+}$ (middle), and right-circularly, $\varphi _{-}$ (right) polarized components of the condensate for the polariton states $| \uparrow \rangle$, $| \downarrow \rangle$ and $| 0 \rangle$ indicated in (**a**). The black drops in the middle and right columns show the polariton currents in the corresponding polarizations. (**c**) The change of the dependence of the energy functional, ${\mathscr {E}}(\ell )$, on OAM with the displacement of the pump spot. The insert in (**c**) shows the change of the energy, *E*, of the ground states of the polariton condensate corresponding to the minima of the functional ${\mathscr {E}} (\ell )$ with increasing $r_{\text {p}}$.

The rotational symmetry break of the system is contained in the interaction part of the Hamiltonian which includes both polariton-polariton interaction and interaction of polaritons with the optically-induced reservoir of excitons. We consider the partially polarized pump described by the vector $|P \rangle = 0.5 P({\mathbf {r}},{\mathbf {r}}_{\text {p}}) (1 + p - \eta p, 1 - p + \eta p)^{\text {T}}$, where $\eta$ defines the unpolarized fraction of the pump. The parameter *p* varying from $-1$ to $+1$ characterizes polarization of the rest of the pump which is right-circularly (left-circularly) polarized when $p=1$ ($-1$). $P({\mathbf {r}},{\mathbf {r}}_{\text {p}})$ is responsible for the spatial distribution of the pump, which is taken in the Gaussian form shifted by ${\mathbf {r}}_{\text {p}}$ from the center of the pillar. Since the pump is rotationally symmetric around the point ${\mathbf {r}}_{\text {p}}$, the azimuthal coordinate of the shift does not affect the orbital degree of freedom, and without loss of generality we can limit ourselves to considering ${\mathbf {r}}_{\text {p}} = (r_{\text {p}},0)$. The parameters $r_{\text {p}}$ and *p* are the control parameters of the circular polariton fluid.

The spin-polarized reservoir of excitons emerges predominantly under the pump spot. The displacement of the pump within the pillar plane causes the displacement of the cloud of the reservoir excitons which contributes to the effective potential. This results in the azimuthal dependence of the depth of the trap for polaritons. The resulting potential acquires the ${\mathtt {Z}}_2$ symmetry ($x \rightarrow -x$). The azimuthal interaction Hamiltonian takes the following form:

2 $$\begin{aligned} {\hat{H}}_{\text {int}} = \sum _{j=\pm } \left[ U_1^j (\theta ) + U_2^j (\theta ) |\Psi _j (t,\theta )|^2 \right. \left. + U_3^{-j} (\theta ) |\Psi _{-j} (t,\theta )|^2 \right] {\check{\sigma }} _j, \end{aligned}$$

where we have introduced the notation ${\check{\sigma }}_{\pm } = \frac{1}{2} ({\check{\sigma }}_0 \pm {\check{\sigma }}_z)$. The functions $U_{1,2,3} ^{\pm } (\theta )$ derived in Methods characterize the azimuthal dependence of the contribution from the polariton-polariton and polariton-exciton interactions to the effective potential. Interactions of polaritons both with the same and opposite spins are taken into account.

In further consideration, we perform a series of numerical experiments reproducing realistic evolution of a spinor exciton-polariton condensate characterized by the Hamiltonians (1) and (2). We take into account non-conservative processes of gain and loss characteristic to realistic experimental conditions. The model used to describe the evolution of the spinor $|\Psi \rangle = \left[ \Psi _+(t,\theta ), \Psi _- (t,\theta ) \right] ^{\text {T}}$ is discussed in detail in Methods.

It is convenient to characterize azimuthal polariton currents in an inhomogeneous potential by the average OAM per particle $\ell = N^{-1} \langle \Psi | {\hat{L}} {\check{\sigma }}_0|\Psi \rangle$, where $N=\langle \Psi |\Psi \rangle$ is the population of the polariton state. The spin-orbit interaction breaks the rotational symmetry and assigns chirality to the system. In these conditions, clockwise ($\circlearrowright$), corresponding to $\ell <0$, and anti-clockwise ($\circlearrowleft$), corresponding to $\ell >0$, polariton currents can emerge in different polarizations. These circumstances allow us to characterize the spinor polariton condensate by the vector $| \ell \rangle = | \ell _+ \ell _- \rangle$ and to limit our consideration to three persistent current states which are co-winding anti-clockwise ${| \uparrow \rangle = | \circlearrowleft \circlearrowleft \rangle }$, co-winding clockwise ${| \downarrow \rangle = | \circlearrowright \circlearrowright \rangle }$ and counter-winding ${| 0 \rangle = | \circlearrowright \circlearrowleft \rangle }$ states. $\ell _{\pm }$ are OAMs in the corresponding polarizations.

### The spontaneous symmetry breaking of the polariton current states

In the first series of numerical experiments, we trace the variation of $\ell$ characterizing the polariton condensate in the steady state with the change of the displacement of the pump spot $r_{\text {p}}$ under the linearly-polarized pump ($p=0$). The resulting dependence is presented in Fig. 2a. At small displacement, $r_{\text {p}}$, the polariton condensate is in the state $| 0 \rangle$ characterized by zero OAM ($\ell = 0$). With the increasing $r_{\text {p}}$ the polariton condensate undergoes the transition to the $|\ell \ne 0 \rangle$ state, herewith the current states $| \uparrow \rangle$ and $| \downarrow \rangle$ which differ by the direction of the currents in both polarization emerge stochastically with equal probabilities. The insert in Fig. 2a shows the OAM states of the polariton condensate in multiple numerical experiments carried out under similar conditions ($r_{\text {p}} = 1.4 \, \upmu \text {m}$ and $p=0$). The distribution of the results between the $| \uparrow \rangle$ and $| \downarrow \rangle$ states is random and homogeneous.

One should mention the polarization properties of the spinor polariton condensate in the steady state. The polarization is characterized by the Stokes vector ${\mathbf {s}} = (s_x,s_y,s_z)$ with the components ${{s_j = N^{-1} \langle \Psi |{\check{\sigma }}_j| \Psi \rangle }}$, where ${j=x,y,z}$, and $|{\mathbf {s}}| = 1$. The populations of the left- and right-circularly polarized components in the $|0\rangle$ state are equal while in the $|\ell \ne 0\rangle$ state they are close to each other such that the circular polarization degree does not exceed 5%.

Figure 2b shows examples of the different states of polariton condensates. In the $| 0 \rangle$ state (lower panels in Fig. 2b) the density distribution of the polariton condensate is symmetrical about the axis of the displacement of the pump spot. The left- and right-circularly polarized components contain vortex and antivortex around the pillar indicating the emergence of the spinor polariton currents in the corresponding directions. The vorticity in different polarization components is characterized by the winding numbers $m _{\pm }$ found as $m _{\pm } = (2 \pi )^{-1} \int \partial _{\theta } \phi _{\pm } (\theta ) d \theta$, where $\phi _{\pm } (\theta )$ are the azimuthal dependencies of the phases of the corresponding polarization components. $m _{\pm }$ quantize and play the role of topological charges. In the $| 0 \rangle$ state, the phases of the circular polarization components of the polariton condensate makes a full turn around the pillar which results in the values of the winding numbers $m_{\mp } = \pm 1$.

In the $|\ell \ne 0 \rangle$ state, the density distribution loses its axial symmetry, herewith the distributions in the $| \uparrow \rangle$ and $| \downarrow \rangle$ states are mirror-symmetrical to each other. The phases of the two circular polarization components depend on the azimuthal angle in the same way, so that two spin-polarised superfluid currents are parallel. This is in contrast to the $| 0 \rangle$ state where we observe antiparallel circular currents. Another peculiarity of the states shown in the upper and middle panels in Fig. 2b is that although azimuthal polariton currents are present in both polarizations, only one of the polarizations contains a vortex. In the discussed case, at large displacement of the pump spot the impact of particle-particle interactions on the azimuthal polariton behavior is considerable in comparison with the role of SOI. In the co-winding regime, the polariton current in one of the polarizations keeps its direction imposed by the spin-orbit interaction, while the current in the opposite polarization changes its direction. In this case, for the former state, the spin-orbit interaction and particle-particle interaction effects cooperate in the formation of the current state and support formation of a vortex. For the latter one they tend to compensate each other, that prevents formation of a vortex state. In particular, in the $| \uparrow \rangle$ state the winding numbers are $m_+ = 0$ and $m_- = +1$, so the vortex is present in the “−” polarization. In the latter, the overall change of the phase by $+2 \pi$ around the pillar includes the positive rapid variation (jump) of the phase around the dip of the polariton density. In the opposite polarization (“$+$”), the overall change of the phase is zero, and one observes the smooth increase of the phase in the clockwise direction along the ridge of the condensate compensated by a step-like decrease of the phase at the dip of the density. The situation is opposite in the $| \downarrow \rangle$ state which is characterized by $m_+ = -1$ and $m_- = 0$, and which exhibits an antivortex in the “$+$” polarization.

The spinor polariton condensates possess a half-moon-shape density distribution due to the broken rotational symmetry. Scalar half-moon condensates have been studied in Ref.^43,44^. The azimuthal variation of the condensate density in addition to the variation of its phase results in fractional values of the average OAM. For the states ${|\uparrow \rangle }$ and ${| \downarrow \rangle }$ illustrated in Fig. 2a the values of $\ell$ are about $\pm 0.72$. The black drops in the distribution of the phases of the condensates in Fig. 2b illustrate the steady state polariton currents in the corresponding polarizations.

### The interplay of SOI and interactions

For further consideration, it is convenient to refer to the azimuthal spectrum of the spinor polariton condensate characterized by the following decomposition:

3 $$\begin{aligned} \Psi _{\pm } (t,\theta ) = \sum _{m \in {\mathbb {Z}}} \psi _{m} ^{\pm }(t) e ^{\text {i} m \theta }. \end{aligned}$$

To fulfill single-valuedness of the polariton wave function, the azimuthal polariton spectrum possesses a discrete character. $\psi _{m} ^{\pm }(t)$ are the spectral components which characterize vortices with the topological charges *m*. In a similar way, we can decompose the azimuthal distribution of the optical pump, $P(\theta , r_{\text {p}}) = \langle P({\mathbf {r}}, {\mathbf {r}}_{\text {p}}) \rangle _{r}$, as

4 $$\begin{aligned} P (\theta , r_{\text {p}}) = \sum _{m \in {\mathbb {Z}}} P _{m} (r_{\text {p}}) e ^{\text {i} m \theta } \end{aligned}$$

which is a function of the displacement $r_{\text {p}}$.

Both SOI and depending on the optical pump particle interactions affect the azimuthal spectrum of the polariton condensate. Substitution of the decomposition (3) into the SOI part of the Hamiltonian (1) shows that the considered here SOI mechanism couples the non-symmetric spectral components in different polarizations, namely it couples the *m* component in the right-circular polarization ($\psi _m^+$) with the $m+2$ component in the left-circular polarization ($\psi _{m+2}^-$) or equivalently, the *m* component in the left-circular polarization ($\psi _m^-$) with the $m-2$ component in the right-circular polarization ($\psi _{m-2}^+$). This is one of the reasons of the SOI induced chirality of the system.

Another source of mixing spectral components of the polariton condensate is the optical pump which feeds the polariton condensate and defines the shape of the effective potential for polaritons. Mixing of polariton states as a result of particle-particle scattering described by the nonlinear terms in (2) is of a complex nature, which, however, obeys the condition of conservation of the overall vorticity in each act of scattering. The spectral redistribution of polaritons as a result of the described mechanisms is discussed in Methods.

**Figure 3:**
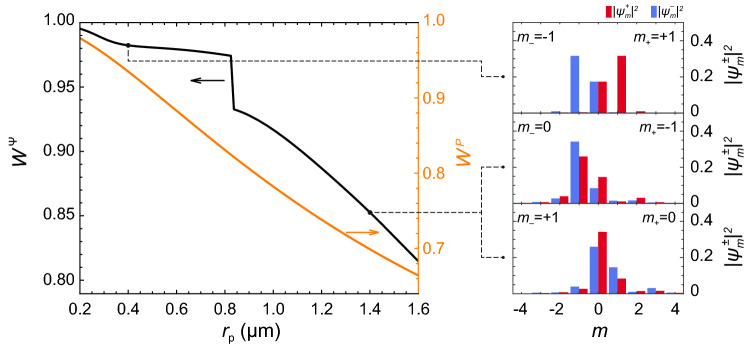
Variation of the spectral width of the wave function. The dependence of the contribution of the population of three central spectral components ($m=0$ and $\pm 1$) of the decomposition (3) to the final polariton state, $W ^{\Psi }$ (black curve), and the contribution of the three central components of the optical pump, $W ^{P}$ (orange curve). The right panels show the populations of the vortex states with the winding numbers *m* from $-4$ to $+4$ contributing to the final polariton states indicated in the main figure.

**Figure 4:**
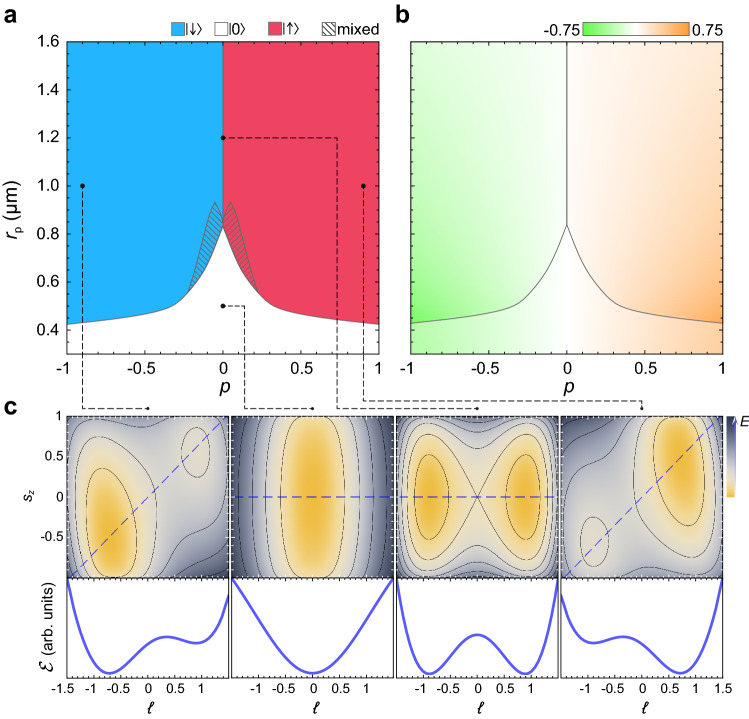
The effect of the pump polarization on the spinor polariton current states. (**a**) The color map showing the variation of the polariton current states $| \uparrow \rangle$, $| \downarrow \rangle$ and $| 0 \rangle$ over the phase plane of the control parameters ${(p,r_{\text {p}})}$. In the shaded regions the polariton condensate occupies $| \uparrow \rangle$ and $| 0 \rangle$ (pink shaded) or $| \downarrow \rangle$ and $| 0 \rangle$ (blue shaded) states in different numerical experiments. (**b**) The dependence of the degree of circular polarization of the polariton condensate, $s_z$, as a function of $r_{\text {p}}$ and *p*. (**c**) Variation of the energy functional, ${\mathscr {E}}$, with the change of the parameters $\ell$ and $s_z$ for the polariton states indicated in (**a**). The upper panels show variation of ${\mathscr {E}}$ in the plane $(\ell ,s_z)$. The lower panels show variation of ${\mathscr {E}}$ with the change of $\ell$ along the dashed line in the corresponding upper panels.

In Fig. 3 we show the contribution of the three central components of the decomposition (3) characterized by ${m=0}$ and ${\pm 1}$ to the final polariton state, $W^{\Psi } = N^{-1} \sum _{j\pm } \sum _{m=0,\pm 1} | \psi _m^{j}|^2$, as well as the fraction of the same components of the pump (4) in the whole spectrum, $W^P = \left( \sum _{m=0,\pm 1} P_m\right) \left( \sum _{m \in {\mathbb {Z}}} P _{m} \right) ^{-1}$, as functions of the pump displacement $r_{\text {p}}$. If the displacement $r_{\text {p}}$ of the pump spot is small, the azimuthal distribution $P(\theta , r _{\text {p}})$ is described by the function $P_0 + P_1 \cos (\theta )$ with a great accuracy. In this limit, the parameter $W^P$ is close to one. This means that the central states with $m=0,\pm 1$ are predominantly pumped, and their evolution determines the evolution of the entire polariton condensate. The induced by interactions effective potential is weakly modulated in the azimuthal direction, and its contribution to the azimuthal behavior of the polariton condensate is dominated by SOI. The SOI-induced chirality determines formation of the polariton $| 0 \rangle$ state with counter-winding currents in opposite circular polarizations. With the increasing displacement $r_{\text {p}}$ the contribution of the spectral components $P_m$ of the pump characterized by $|m|>1$ monotonically increases (while $W^P$ decreases), which brings the higher spectral components of the wave function (3) into play. This stimulates redistribution of the azimuthal spectrum of the polariton condensate due to interactions. If the SOI contribution to the mixing process is dominated by the pump-induced mixing, the system switches to the state with $\ell \ne 0$. At the critical value of $r_{\text {p}}$ the contribution of the states with $|m|>1$ abruptly increases (see the kink in the black curve in Fig. 3 around $r_{\text {p}} = 0.85 \, \upmu \text {m}$) followed by the monotonic decrease of $W^{\Psi }$ with the increase of $r_{\text {p}}$. The relative contributions of various integer angular momentum states into the polariton condensate wave function in different current states are shown in the right column in Fig. 3.

### The energy of the polariton current state

Averaging the total Hamiltonian ${\hat{H}} = {\hat{H}}_0 + {\hat{H}}_{\text {int}}$, we can find the mean-field energy of the polariton condensate, $E = \langle \Psi | {\hat{H}} |\Psi \rangle$, corresponding to the solutions presented in Fig. 2a. The ground-state energy corresponds to the minima of the energy functional written in the general form as

5 $$\begin{aligned} {\mathscr {E}}(\ell ) = {\mathscr {E}} _{\text {SOI}} (\ell ) + {\mathscr {E}} _{\text {int}} (\ell ), \end{aligned}$$

that is a function of the order parameter $\ell$. The first and second terms in (5) are responsible for the contribution of SOI and interactions, respectively. As we have discussed above, the linear polarization of the optical pump ($p=0$) is inherited by the polariton condensate, so the components of the classical pseudospin vector take values $s_z \simeq 0$ and $s_x \simeq 1$. The Hamiltonian ${\hat{H}}_0$ in (1) contains an explicit contribution of the OAM operator. The structure of the functional ${\mathscr {E}} _{\text {SOI}} (\ell )$ repeats the structure of ${\hat{H}}_0$, and at $p=0$ it takes the following form:

6 $$\begin{aligned} {\mathscr {E}} _{\text {SOI}} (\ell ) = c_0 + c_1 \ell ^2, \end{aligned}$$

where $c_0$ and $c_1$ are the constants emerging after the integration over $\theta$. In the case of the rotationally-symmetric trapping potential, where the contribution of interactions into the azimuthal polariton behavior of polaritons is reduced, the parameters $\ell$ and *m* merge, and Eq. (6) describes the quantized parabolic spectrum of the annular polariton states.

The contribution of the interactions is determined by the azimuthal distribution of the polariton condensate as well as of the exciton reservoir, and in general case it should be calculated self-consistently. However, the energy functional ${\mathscr {E}} _{\text {int}}(\ell )$ to a good approximation can be written in the following form (see Methods for the approximation):

7 $$\begin{aligned} {\mathscr {E}} _{\text {int}}(\ell ) = E_{\text {J}} \cos (\zeta \ell ). \end{aligned}$$

As one can see, the interaction energy, ${\mathscr {E}} _{\text {int}}(\ell )$, decreases with increasing OAM and it is minimized at $\ell \ne 0$. This can be explained as follows. The optically-excited reservoir of incoherent excitons plays two important for our problem roles. First, it acts as a source of polaritons for the condensate. Second, it is responsible for the energy blueshift due to the repulsive polariton-exciton interaction. The spatially localized exciton reservoir cloud forms a potential maximum providing the system with the gradient of the effective potential. Polaritons emerging within the reservoir spot tend to move away from it along the potential gradient. This causes the decrease in the overlap of the condensate and the reservoir, which results in the reduction of the reservoir-induced blueshift. This scenario is valid in our system if the exciton reservoir is shifted from the center of the pillar which is why it causes the azimuthal modulation of the effective potential. In the azimuthally symmetric annular geometry, the interaction energy of the polariton condensate is not affected by its OAM.

The effective potential (7) is playing the same role in quantization of the energy of a polariton condensate as the effective double-well potential induced by Josephson junctions plays for quantization of energy of superconducting flux qubits^48,49^ The central potential barrier isolates the two OAM states, $| \uparrow \rangle$ and $| \downarrow \rangle$, from each another. It is important to note that the energy constant $E_{\text {J}}$ does not depend on the order parameter $\ell$, herewith it can be tuned by varying the control parameter of the displacement of the pump $r_{\text {p}}$. $\zeta$ is the parameter, responsible for the control of the orbital momentum of the condensate, $\ell$, due to the inhomogeneity of the azimuthal distribution of the density of the polariton condensate. The increase of $E_{\text {J}}$ with $r_{\text {p}}$ results in the symmetry breaking of the polariton condensate manifested in the switching from the single counter-winding state to the two degenerate co-winding states. This symmetry breaking is evidenced by the set of numerical results summarized in Fig. 2a. In Fig. 2c we show schematically the variation of the energy-OAM dependence (5) with the change of the control parameter $r_{\text {p}}$. The minima of the presented dispersions ${\mathscr {E}} (\ell )$ correspond to the energies, *E*, of the ground states of the polariton condensate which are the $| \uparrow \rangle$, $| \downarrow \rangle$ and $|0 \rangle$ states. In the insert in Fig. 2c the variation of *E* with the change of $r_{\text {p}}$ is shown.

### The effect of the polarization of the pump

We now investigate the effect of the polarization of the pump onto the symmetry breaking of the polariton current state. The color map in Fig. 4a shows the polariton current states in the plane of the control parameters $(p, r_{\text {p}})$. The corresponding circular polarization degree characterized by the Stokes vector component $s_z$ is shown in Fig. 4b. The circular polarization of the pump partially transfers to the polariton condensate. The imposition of circular polarization does not cancel the switching between the $|0\rangle$ and $|\ell \ne 0 \rangle$ states, herewith it reduces the critical value of the displacement $r_{\text {p}}$ characteristic of this switching. In addition, the circular polarization of the pump removes the degeneracy of energies of the $| \uparrow \rangle$ and $| \downarrow \rangle$ states in the $\ell \ne 0$ states. Namely, under the predominant right-circular polarization (“$+$”), the polariton condensate occupies the $| \downarrow \rangle$ state and supports clockwise currents in both polarizations. In contrast, under the left-circularly polarized pump (“$+$”), the condensate occupies the $| \uparrow \rangle$ state characterized by anti-clockwise currents. The choice of the direction of the currents by the polariton condensate at different polarizations of the pump is determined by the SOI-induced symmetry breaking discussed in previous sections. The color map in Fig. 4a contains the transition regions of the mixed phases where both $| 0 \rangle$ and $| \ell \ne 0 \rangle$ states emerge spontaneously. Herewith, the separation of the states with the co-winding clockwise and anti-clockwise polariton currents depending on the polarization of the pump is maintained.

The spin (polarization) degree of freedom contributes to the energy of the polariton condensate, ${\mathscr {E}} (\ell ) \rightarrow {\mathscr {E}} (\ell , s_z)$. The SOI part of the energy functional keeps its form (6) with the constants modified as follows: ${c_0 \rightarrow c_0 s_x}$ and ${c_1 \rightarrow c_1 +c_2 s_x}$. The structure of the functional ${\mathscr {E}} _{\text {SOI}} (\ell , s_z)$ repeats the structure of the Hamiltonian (1) after a unitary transformation ${\check{A}} {\hat{H}}_0 {\check{A}}^{-1}$, where ${\check{A}} = \cos (\theta ) {\check{\sigma }}_0 +\text {i} \sin (\theta ) {\check{\sigma }}_z$ is the rotation matrix. The spin vector components are linked to each other by $s_x = (1 - s_z^2)^{1/2}$.

The contribution of the spin to the interaction part (6) is more complicated, which, nevertheless, can be revealed by fitting the numerical results in Fig. 4a. The phenomenological prefactor ${E_{\text {J}} (\ell , s_z)}$ now depends on $s_z$, which describes the reduction of the critical value of the displacement $r_{\text {p}}$ with the increase of the contribution of the circular polarization of the pump. The argument of the cosine, $\zeta \ell$, transforms to $\zeta \ell + \zeta _1 p + f(p) s_z$, where the second term is responsible for lifting the degeneracy of the states $|\uparrow \rangle$ and $| \downarrow \rangle$ with the increase of |*p*|. The last term is responsible for the circular polarization of the polariton condensate in the current state $|\ell \rangle$. *f*(*p*) is the even function of the control parameter *p*. In Fig. 4c we represent the typical maps showing the variation of the energy functional on the phase plane $(\ell , s_z)$ for the selected polariton states indicated in Fig. 4a. The color maps are supplemented by the variation of the energy functional along the line on the plane $(\ell , s_z)$ linking its minima for the $| \ell \ne 0 \rangle$ state and along the line $s_z=0$ for the $| 0 \rangle$ state. The color maps Fig. 4a, b correspond to the minimization of the functional with respect to the variables $\ell$ and $s_z$.

## Discussion

In this manuscript, we have predicted the spontaneous symmetry breaking in the system of persistent azimuthal currents in the annular spinor exciton-polariton condensate under the effects of SOI and particle-particle interactions. An approach to the trapping of polaritons bases on combining the stationary confinement potential from the cylindrical micropillar and the optically induced core repulsive potential makes it possible to control the symmetry of the effective trapping potential. Displacing the pump spot from the center of the pillar reduces the symmetry of the trap from the rotational symmetry to the axial (${\mathtt {Z}}_2$) symmetry, while the SOI effect endows the system with chirality.

The symmetry breaking occurs with the increase in the displacement of the pump where the effect of particle-particle interactions starts dominating the contribution of SOI to the azimuthal polariton dynamics. Beyond the transition point, the counter-winding persistent currents of polaritons in opposite circular polarizations are replaced with the co-winding currents, where the direction of the currents can be controlled by the polarization of the optical pump. Average OAM per particle, $\ell$, changes from $\ell = 0$ at small displacement to $\ell \ne 0$ with the increasing displacement.

The newly found polariton current states characterised by fractional OAM can be compared with half-vortices^34,35^ and spin-mediated half-quantum circulations^33^. The states of a spinor condensate studied in this work are characterized by the presence of azimuthal polariton currents in both circular polarizations, however only one of them possesses vorticity. The currents are supported by the azimuthal inhomogeneity of the trapping potential which also leads to the half-moon shape of the polariton condensate. The dip in the azimuthal density distribution of polariton allows for controlling variation of the phase of the condensate, so that the condition of the single-valuedness of the wave function is fulfilled.

The symmetry breaking is characterized by the change of the shape of the energy functional ${\mathscr {E}}(\ell )$ from a single-well to a double-well. For description of the spinor polariton current states in the considered system, an approach analogous to one successfully employed for the description of flux qubits in superconducting circuits containing Josephson junctions can be used. The fundamental similarity between half-moon-shape polariton condensates and superconducting flux qubits confirms the high potential of polariton condensates for realisation of qubits and quantum networks as discussed in^50^.

We expect that the effects discussed in this manuscript are characteristic to the whole class of polaritonic systems confined in annular potentials regardless the origin of these potentials. In particular, as an alternative geometry of the possible experiment we suggest optically induced ring traps^51–53^. An additional option for controlling the polariton state in this geometry is by manipulating the azimuthal distribution of the polarization of the optical pump.

## Methods

### Generalized Gross–Pitaevskii equation with a spin-resolved reservoir

The polariton condensate is described by gGPE for the spinor $|\Phi \rangle = [\Phi _+ (t,{\mathbf {r}}), \Phi _- (t,{\mathbf {r}})]^{\text {T}}$, where $\Phi _{\pm } (t,{\mathbf {r}})$ are the wave functions of the left- and right-circularly polarized polaritons:

8 $$\begin{aligned} \text {i} \hbar |\Phi \rangle = \left[ {\hat{T}} + {\hat{V}} \right] |\Phi \rangle +\frac{\text {i}\hbar }{2} \left[ {\hat{R}}_{\text {in}} - \gamma {\check{\sigma }}_0 \right] |\Phi \rangle , \end{aligned}$$

where the operator

9 $$\begin{aligned} {\hat{T}} = \frac{\hbar ^2{\hat{k}}^2}{2M} {\check{\sigma }}_0 + \frac{\hbar \Delta }{2}\left[ ({\hat{k}}_x^2 - {\hat{k}}_y^2){\check{\sigma }}_x + 2{\hat{k}}_x {\hat{k}}_y {\check{\sigma }}_y \right] \end{aligned}$$

is responsible for the kinetic energy of polaritons taking into account the TE-TM splitting. $\hat{{\mathbf {k}}} = ({\hat{k}}_x,{\hat{k}}_y) = (-\mathrm {i} \partial _x,-\mathrm {i} \partial _y)$ is the wave vector operator.

The potential energy operator is given by

10 $$\begin{aligned} {\hat{V}} = V_{\text{c}}(r) {\check{\sigma }}_0 + \sum _{j = \pm }\left[ \alpha _1 |\Phi _{j} (t,{\mathbf {r}})|^2 + \alpha _2 |\Phi _{-j} (t,{\mathbf {r}})|^2 \right. \left. +\alpha _{\text {R}1} n_{\text {R} \, j}(t,{\mathbf {r}}) + \alpha _{\text {R}2} n_{\text {R} \,(-j)}(t,{\mathbf {r}}) \right] {\check{\sigma }}_j , \end{aligned}$$

where *V*_c_(*r*) is the cylindrical stationary potential. The second term in (10) is responsible for repulsive interaction of polaritons within the condensate and polaritons with the reservoir of incoherent excitons. The interaction is spin-selective. $\alpha _1$ and $\alpha _2$ are the interaction constants of polaritons with the same and opposite spins, respectively. $\alpha _{\text {R}1}$ and $\alpha _{\text {R}2}$ are the polariton-reservoir interaction constants. $n_{\text {R}\pm } (t,{\mathbf {r}})$ are the particle densities in two spin components of the reservoir.

The last term in (8) describes the balance of gain and loss in the polariton condensate. The operator

11 $$\begin{aligned} {\hat{R}} _{\text {in}} = R \left[ n_{\text {R}+}(t,{\mathbf {r}}) {\check{\sigma }} _+ + n_{\text {R}-}(t,{\mathbf {r}}) {\check{\sigma }}_- \right] \end{aligned}$$

is responsible for the stimulated inflow of particles from the reservoir with the rate *R*. $\gamma$ is the polariton decay rate.

### The reservoir of incoherent excitons

The polariton state is fed from the spin-resolved reservoir described by the spinor $|n_{\text {R}}\rangle = [n_{\text {R}+} (t,{\mathbf {r}}), n_{\text {R}-} (t,{\mathbf {r}})]^{\text {T}}$ obeying the following rate equation:

12 $$\begin{aligned} \partial _t | n_{\text {R}}\rangle = |P \rangle -(\gamma _{\text {R}} {\check{\sigma }} _0 + {\hat{R}} _{\text {out}}) | n_{\text {R}}\rangle , \end{aligned}$$

where $|P \rangle$ describes the spin-resolved non-resonant optical pump:

13 $$\begin{aligned} |P \rangle = P({\mathbf {r}}) \left[ \frac{1}{2}\eta \begin{pmatrix} 1 \\ 1 \end{pmatrix} + (1- \eta ) \begin{pmatrix} p_+\\ p_- \end{pmatrix} \right] = \frac{1}{2}P({\mathbf {r}}) \begin{pmatrix} 1 + p - \eta p\\ 1 - p + \eta p \end{pmatrix} . \end{aligned}$$

Polarization of the pump is partially lost during relaxation of photoexcited excitons to the reservoir state. $\eta$ is the fraction of the pump with the lost polarization which equally pumps both spin components of the reservoir. $p_{\pm }$ describe polarization of the optical pump, $p_+ + p_- = 1$. Another option to characterize the polarization of the pump used in the main text is the single parameter *p* linked to $p_{\pm }$ as follows: $p_+ = 0.5(1+p)$ and $p_- = 0.5(1-p)$. $P({\mathbf {r}})$ is responsible for the spatial distribution of the pump taken in the Gaussian form:

14 $$\begin{aligned} P({\mathbf {r}}) \propto \exp \left[ - \frac{(x - x _{\text {p}})^2 + (y - y _{\text {p}})^2}{2w_{\text {p}}^2} \right] , \end{aligned}$$

shifted from the center of the pillar by ${\mathbf {r}}_{\text {p}} = (x_{\text {p}},y_{\text {p}})$ (${\mathbf {r}}_{\text {p}} = (r_{\text {p}},\theta _{\text {p}})$ in polar coordinates). $w_{\text {p}}$ is the width of the pump. We represent the pump $P({\mathbf {r}})$ as a combination of the azimuthally symmetric component and the symmetry-breaking addition as $P({\mathbf {r}}) = P_{\text {s}} (r) + \delta P({\mathbf {r}})$, where $P_{\text {s}} (r) = P({\mathbf {r}}) |_{{\mathbf {r}}_{\text {p}} \rightarrow (0,0)}$. $\gamma _{\text {R}}$ is the reservoir decay rate. The operator ${\hat{R}} _{\text {out}}$ describes an outflow of particles from the reservoir. It is obtained from ${\hat{R}} _{\text {in}}$ by replacing $n_{\text {R}\pm } (t,{\mathbf {r}}) \rightarrow |\Phi _{\pm } (t,{\mathbf {r}})|^2$.

We eliminate the reservoir from Eq. (12) taking it in the form

15 $$\begin{aligned} | n_{\text {R}}\rangle = \frac{| P\rangle }{\gamma _{\text {R}} + {\hat{R}} _{\text {out}}} \approx \frac{1}{\gamma _{\text {R}}} \left( 1 - \frac{1}{\gamma _{\text {R}}} {\hat{R}}_{\text {out}} \right) | P\rangle . \end{aligned}$$

### Reduction to 1D

We split radial and azimuthal components of the polariton wave function representing it as follows: $| \Phi \rangle = \Upsilon (r) \exp (-\text {i} E_r t / \hbar ) |\Psi \rangle$, where $|\Psi \rangle = [ \Psi _{+} (t, \theta ), \Psi _{-} (t, \theta ) ]^{\text {T}}$ is the azimuthal wave function component, $\Upsilon (r)$ is the radial wave function component. We limit ourselves to the lowest radial mode obeying the equation

16 $$\begin{aligned} E_r \Upsilon (r) = \left[ -\frac{\hbar ^2 }{2 M} \left( \partial _{rr}^2 +\frac{1}{r} \partial _r \right) + V_{\text {c}}(r) \right. \left. + (\alpha _{\text {R}1} + \alpha _{\text {R}2})\frac{\eta P _{\text {s}}(r) }{2\gamma _{\text {R}} } \right] \Upsilon (r) +\mathrm {i} \frac{\hbar }{2}\left[ \frac{\eta R P_{\text {s}}(r)}{2 \gamma _{\text {R}}} - \gamma \right] \Upsilon (r). \end{aligned}$$

Averaging Eq. (8) over $\Upsilon (r)$ we obtain the following equation for the azimuthal wave function component:

17 $$\begin{aligned} \text {i} \hbar \partial _t |\Psi \rangle = \left[ {\hat{H}}_0 + {\hat{H}}_{\text {int}} + \text {i}{\hat{I}} \right] |\Psi \rangle . \end{aligned}$$

where the Hamiltonians ${\hat{H}}_0$ and ${\hat{H}}_{\text {int}}$ are given by (1) and (2), respectively. The operator ${\hat{I}}$ is responsible for the balance of the gain and loss in the azimuthal direction. It repeats the structure of the part of the Hamiltonian ${\hat{H}}_{\text {int}}$ for the interaction of polaritons with the same spins, and it can be obtained from it by replacing $U_{1,2}^{\pm } (\theta ) \rightarrow I_{1,2}^{\pm } (\theta )$ and $U_3^{\pm } (\theta ) \rightarrow 0$. The functions $U_j^{\pm } (\theta )$ and $I_j^{\pm } (\theta )$ are given as follows:

18a $$\begin{aligned} U_1 ^{\pm } (\theta )= & {} \left\langle \Upsilon (r) \left| \left( \alpha _{\text {R}1} + \alpha _{\text {R}2} \right) \frac{\eta \delta P({\mathbf {r}})}{2 \gamma _{\text {R}}} \right. \right. + \left( \alpha _{\text {R}1} p_{\pm } + \alpha _{\text {R}2} p_{\mp } \right) \left. \left. \frac{(1-\eta ) P({\mathbf {r}})}{\gamma _{\text {R}}} \right| \Upsilon (r) \right\rangle , \end{aligned}$$

18b $$\begin{aligned} I_1 ^{\pm } (\theta )= & {} \frac{\hbar R}{2 \gamma _{\text {R}}} \left\langle \Upsilon (r) \left| \frac{\eta \delta P({\mathbf {r}})}{2} \right. \right. \left. \left. + (1-\eta )p_{\pm } P({\mathbf {r}}) \right| \Upsilon (r) \right\rangle , \end{aligned}$$

18c $$\begin{aligned} U_{2,3} ^{\pm } (\theta )= & {} \left\langle \Upsilon (r) \left| \left\{ \alpha _{1,2} -\frac{\alpha _{\text {R}1,2} R P({\mathbf {r}}) }{\gamma _{\text {R}}^2} \right. \right. \right. \left. \left. \left. \left[ \frac{1}{2}\eta +(1-\eta )p_{\pm } \right] \right\} |\Upsilon (r)|^2 \right| \Upsilon (r) \right\rangle , \qquad \end{aligned}$$

18d $$\begin{aligned} I_2 ^{\pm } (\theta )= & {} - \frac{\hbar R^2}{2\gamma _{\text {R}}^2} \left[ \frac{1}{2}\eta +(1-\eta )p_{\pm } \right] \left\langle \Upsilon (r) \left| P({\mathbf {r}}) |\Upsilon (r)|^2 \right| \Upsilon (r) \right\rangle . \end{aligned}$$

In (18c) the indices “±” and the numeric indices are independent of each other.

**Figure 5:**
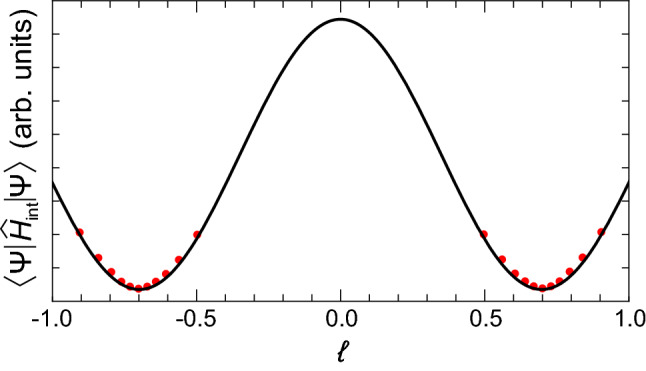
Approximation of the interaction energy. The parametric dependence of the interaction energy $\langle \Psi | {\hat{H}} _{\text {int}} | \Psi \rangle$ on OAM of the polariton condensate driven by supplemental rotation. Red dots indicate solutions of Eq. (17) with the Hamiltonian ${\hat{H}}_{0}$ supplemented with the rotation term $\hbar \omega _{\text {rot}} {\hat{L}} {\check{\sigma }}_0$. The black curve is the fit with the function $\cos ( \zeta \theta )$. The shift of the pump spot is taken $r_{\text {p}} = 1.1 \, \upmu \text {m}$, the polarization of the pump is linear ($p=0$).

### Projected gGPE

For the next step, we use the decomposition (3) for the wave function to represent it as a linear combination of vortex states characterized by winding numbers *m*. We also decompose the coefficients $U_j^{\pm } (\theta )$ and $I_j^{\pm } (\theta )$ as follows:

19a $$\begin{aligned} U_j ^{\pm } (\theta )= & {} \sum _{m \in {\mathbb {Z}}} U _{j,m} ^{\pm } e ^{\text {i} m \theta }, \end{aligned}$$

19b $$\begin{aligned} I_j ^{\pm } (\theta )= & {} \sum _{m \in {\mathbb {Z}}} I _{j,m} ^{\pm } e ^{\text {i} m \theta }. \end{aligned}$$

Substituting (3) and (19) into (17), we arrive at the following system of the first-order differential equations for the coefficients $\psi _{m} ^{\pm } (t)$:

20 $$\begin{aligned}&\mathrm {i} \hbar \partial _t \psi _{m} ^{\pm } = \frac{\hbar ^2}{2 M \rho ^2} m^2 \psi _m ^{\pm } + \sum _{m' \in {\mathbb {Z}}} \left( U_{1,m'}^{\pm } + \text {i} I_{1,m'}^{\pm } \right) \psi _{m-m'} - \frac{\hbar \Delta }{2} \left[ \frac{(m \pm 2)^2 }{\rho ^2} \mp \frac{2 (m \pm 2)}{\rho _2^2} + \frac{1}{\rho _1^2} \right] \psi _{m \pm 2} ^{\mp } \nonumber \\&\quad + \sum _{m' \in {\mathbb {Z}}} \, \sum _{m'' \in {\mathbb {Z}}} \, \sum _{m''' \in {\mathbb {Z}}} \sum _{m'''' \in {\mathbb {Z}}} \delta _{m+m'''', m'+m''+m'''} \left[ \left( U _{2,m'}^{\pm } + \text {i} I _{2,m'}^{\pm } \right) ( \psi _{m''''} ^{\pm })^* \psi _{m'''}^{\pm } \right. \left. + U _{3,m'}^{\mp } ( \psi _{m''''} ^{\mp })^* \psi _{m'''}^{\mp } \right] \psi _{m''}^{\pm }. \end{aligned}$$

The population and OAM per particle are found as follows:

21a $$\begin{aligned} N_{\pm }(t)= & {} \sum _{m \in {\mathbb {Z}}} |\psi _{m} ^{\pm } (t) |^2, \end{aligned}$$

21b $$\begin{aligned} N(t)= & {} N_+(t) + N_-(t), \end{aligned}$$

21c $$\begin{aligned} \ell (t)= & {} \frac{1}{N(t)} \sum _{j=\pm } \sum _{m \in {\mathbb {Z}}} m |\psi _{m} ^{j} (t) |^2. \end{aligned}$$

### Approximation of the interaction component of the energy functional

The polariton condensate tends to minimize its energy during its evolution described by Eq. (17). Reaching a steady state, it occupies the energy minimum and acquires OAM corresponding to it. To reveal how the interaction energy of the polariton condensate characterized by the Hamiltonian ${\hat{H}}_{\text {int}}$ changes with the orbital angular momentum, $\ell$, we supplement the evolution of the condensate with its rotation around the centre of the micropillar. It is described by the term $\hbar \omega _{\text {rot}} {\hat{L}} {\check{\sigma }}_0$ added to the linear Hamiltonian (1). The supplemental rotation makes the condensate increase or decrease its orbital angular momentum which is reflected in the change of the interaction energy. We solve the Gross–Pitaevskii equation (17) with the varying rotational frequency $\omega _{\text {rot}}$ (which can be both negative and positive describing rotation in opposite directions) and trace the change of the interaction energy, $\langle \Psi | {\hat{H}}_{\text {int}} | \Psi \rangle$, and OAM, $\ell$. The parametric dependence of $\langle \Psi | {\hat{H}}_{\text {int}} | \Psi \rangle$ on $\ell$ is shown in Fig. 5 by red dots around two minima corresponding to no supplemental rotation ($\omega _{\text {rot}} = 0$). For the simulations we take the radial pump shift $r_{\text {p}} = 1.1 \, \upmu \text {m}$ and the linearly polarized pump ($p=0$). The obtained dependence can be perfectly fitted by the function ${\mathscr {E}} _{\text {int}}(\ell ) \propto \cos (\zeta \ell )$ around the minima, see the black curve in Fig. 5.

### Values of the parameters

We take the following values of the parameters of the model for simulations. The effective mass of polaritons is $M = 5 \times 10^{-5} m_{\text {e}}$, where $m_{\text {e}}$ is the free electron mass. The polariton and reservoir decay rates are $\gamma = 0.02$ ps$^{-1}$ and $\gamma _{\text {R}} = 0.025$ ps$^{-1}$, respectively. The scattering rate is $\hbar R=0.05\, \text {meV} \, \upmu \text {m}^2$. The nonlinearity coefficients are $\alpha _1 = \alpha _{\text {R}1}/2 = 3 \, \upmu \text {eV} \, \upmu \text {m}^2$, $\alpha _2 = \alpha _{\text {R}2}/2=-0.1 \alpha _1$. The TE-TM splitting constant is $\hbar \Delta = 150 \, \upmu \text {eV} \, \upmu \text {m}^2$. The pump width is $w_{\text {p}} = 2 \, \upmu \text {m}$. The diameter of the pillar is $25\, \upmu \text {m}$. The nonpolarized fraction is $\eta = 0.7$.
